# Linking Reproduction and Survival Can Improve Model Estimates of Vital Rates Derived from Limited Time-Series Counts of Pinnipeds and Other Species

**DOI:** 10.1371/journal.pone.0077389

**Published:** 2013-11-12

**Authors:** Brian C. Battaile, Andrew W. Trites

**Affiliations:** Marine Mammal Research Unit, Fisheries Centre, AERL, University of British Columbia, Vancouver, British Columbia, Canada; Institut Pluridisciplinaire Hubert Curien, France

## Abstract

We propose a method to model the physiological link between somatic survival and reproductive output that reduces the number of parameters that need to be estimated by models designed to determine combinations of birth and death rates that produce historic counts of animal populations. We applied our Reproduction and Somatic Survival Linked (RSSL) method to the population counts of three species of North Pacific pinnipeds (harbor seals, *Phoca vitulina richardii* (Gray, 1864); northern fur seals, *Callorhinus ursinus* (L., 1758); and Steller sea lions, *Eumetopias jubatus* (Schreber, 1776))—and found our model outperformed traditional models when fitting vital rates to common types of limited datasets, such as those from counts of pups and adults. However, our model did not perform as well when these basic counts of animals were augmented with additional observations of ratios of juveniles to total non-pups. In this case, the failure of the ratios to improve model performance may indicate that the relationship between survival and reproduction is redefined or disassociated as populations change over time or that the ratio of juveniles to total non-pups is not a meaningful index of vital rates. Overall, our RSSL models show advantages to linking survival and reproduction within models to estimate the vital rates of pinnipeds and other species that have limited time-series of counts.

## Introduction

Significant declines of pinnipeds (seals and sea lions) in the Gulf of Alaska and Bering Sea have focused attention on the need to know birth and survival rates to determine why these populations have declined and why they are not recovering [1]–[3]. In the absence of knowing the reproductive histories and ages at death of known individuals, models are increasingly being used to estimate the most likely combination of age-specific birth and survival rates that could have produced observed population declines (e.g., [4]–[7]). The models are typically fit to annual estimates of total population size, numbers of individuals born, and occasionally the proportions alive by age- or sex-classes. One challenge in fitting age- or stage-structured models to count data to determine vital rates is that the ratio of parameters to data is relatively high, and results in large confidence intervals on the estimated birth and survival rates as well as the fitted model to count data. This shortage of data is often the case for difficult to observe species, and those that are rare and endangered.

Reducing the number of parameters that need to be estimated by age- or stage-structured models could make the method of hind casting vital rates (i.e., modeling past events) more readily applicable to populations with limited time-series of counts. One means of reducing the number of parameters is to combine or link parameters that are correlated or influenced by common factors. Age specific survival and reproductive output are correlated to some degree at many stages―with an increase in reproductive output often closely lagging behind an increase in juvenile survival. Survival and reproduction also tend to peak together as individuals move into their reproductive prime, and then decline as individuals pass the prime of their lives. In the later stages of life, senescence appears to be the most significant process that links survival and reproductive rates.

Senescence is the general decline of an organism's ability to survive and reproduce late in life. The biological study of senescence typically considers survival and reproduction in isolation of one another. However, the two types of senescence are linked [8] through the concept of reproductive value within an evolutionary and ecological framework [9].

The major theories of the evolution of senescence, namely ‘antagonistic pleiotropy’ [10], [11] and the ‘disposable soma’ [12] say little about the relative timing of the two types of senescence beyond a curiosity for the evolution of post reproductive lifespans in females (a specific form of reproductive senescence most often found in social animals) motivated by a desire to understand menopause in women [13]. Among the few field studies on the relative timing of survival and reproductive senescence [14]–[16], reproductive senescence has been noted to begin 6 years after survival senescence in bighorn sheep *Ovis Canadensis* (Shaw, 1804) [14], but co-occurred in subantarctic fur seals *Arctocephalus tropicalis* (Gray, 1972) [16]. It is only recently that the interaction between the two forms of senescence has been addressed [17]–[19]. While evolutionary theories exist to explain the variety in the timing of somatic and reproductive senescence, cases of the relatively close co-occurrence of survival and reproductive senescence may be most parsimoniously explained by the idea that an individual undergoing somatic senescence could not afford the physiological expense of reproduction that would allow it to remain reproductively active until death [20].

A number of studies have documented senescence in pinniped survival [2], [21] and reproduction [22]–[29], or both [5], [16], [30]–[34]—while only a few have failed to detect senescence [27], [35], [36]. In the North Pacific Ocean, three species of pinnipeds have been shown to experience declining fertilities and survival with age [2], [22], [31], [32], [37]—harbor seals, *Phoca vitulina richardii* (Gray, 1864); northern fur seals, *Callorhinus ursinus* (L., 1758); and Steller sea lions, *Eumetopias jubatus* (Schreber, 1776). These three species appear to exhibit a general increase in physiological ‘frailty’ that is associated with somatic senescence and consequently paralleled by decreases in reproductive ability.

Overall, the available life history data suggest there is merit in including senescence in pinniped population dynamics models. Incorporating important life history traits should improve model fit and hence the explanatory power and potential of the model to predict birth and survival rates. However, including additional biological realism such as reproductive senescence into models normally means estimating additional parameters.

In principle, a demographic model that links reproductive and somatic senescence should provide a more parsimonious fit to time series of population counts because they can more accurately describe biological processes while simultaneously reducing the number of estimated variables. Our goal was to thus determine whether model estimates of birth and death rates derived from limited time-series counts of pinnipeds could be improved by mathematically linking reproductive and somatic senescence.

The approach we took to link reproductive and somatic senescence built on the observation by Eberhardt [38] that the Lotka equation could be simplified by assuming that the senescence parameters describing the two portions of the survival and reproductive curves are identical. We thereby populated vital rates in Leslie matrix models by parameterizing reproduction with survival terms that were heavily characterized by senescence. We then compared the results of a number of published and unpublished traditional models with our Reproduction and Somatic Survival Linked (RSSL) model for harbor seals, northern fur seals and Steller sea lions from Alaska. Our expectation was that the RSSL model would be more parsimonious, but that the fit of the model (the difference between model predictions and field counts of animals) would depend upon the strength of the relationship between reproduction and survival as animals entered senescence. Our results show the merits of this modeling approach for understanding the population dynamics of wildlife and the circumstances under which it may or may not be advantageous to link reproduction and survival in population demographic models.

## Materials and Methods

The RSSL model that we developed was based on a modified Leslie matrix. In general we followed the methods of Holmes et al. [5] in formulating the matrix and fitting the model to count data, with some exceptions. Our data came from life tables published for northern fur seals [31] and Steller sea lions [32] and published rates of survival, ovulation and pregnancy for harbor seals [2], [22]. Age specific vital rates were grouped into three categories (juvenile survival, adult survival and adult fertility). We estimated the vital rates by fitting commonly used linear models to describe age-based fertility and survival which were then used to build Leslie matrices. These Leslie matrices were then used to fit to population count data time series by varying the three categories of age specific vital rates over blocks of the available time series.

Our modeling approach had four steps:

1. Estimate maturity and adult survival curves and juvenile survival from life table data.
2. Estimate fertility by multiplying maturity and adult survival curves, and adjusting the maturity curve parameter *b_max_* to fit to pregnancy data.
3. Fill in the Leslie matrix from the results of Steps 1 and 2.
4. Fit the model to time series counts or other population data using maximum likelihood by adjusting adult survival curves (and by association, fertility) and juvenile survival over several distinct time periods corresponding to different population trends.

*Step 1—estimating parameters for vital rate curves.* We began by estimating the proportion of a population that was mature (*M*) using a logistic curve based on the proportion of the population that had ovulated (in the case of harbor seals and sea lions) or were pregnant (in the case of fur seals):
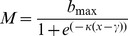
(1)where the asymptote is *b_max_*, gamma (*γ*) specifies the inflection point on the curve (age when 50% of the population is mature), *x* is age in years, and kappa (*κ*)is the approximate age at which the first individuals become mature.

We next estimate survival. A Siler [39] curve
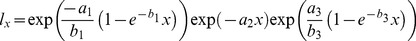
(2a)was fit to the cumulative survivorship (*l_x_*) data for the fur seal and harbor seal data and annual probabilities of adult survival were calculated as
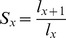
(2b)In the case of the Steller sea lions, we fit their annual survival rates using the Weibull curve [40]

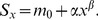
(2c)For both equations, *S_x_* is survival from age *x* to age *x+*1, *a_i_* and *b_i_* are estimated parameters in the Siler, and *m_0_ α* and *β* are estimated in the Weibull. We investigated many mortality functions including the Gompertz, Gompertz-Makeham and logistic for the fur seal and harbor seal data, but none of them provided a satisfactory fit because the Gompertz and Weibull are strictly decreasing functions (with respect to age based annual mortality survival) while our data indicated a continued slight increase in survival through the early years of adulthood prior to the expected decrease associated with senescence. Typically these models are applied at the onset of adulthood which corresponds to the onset of decreasing or stable probabilities of survival, which was not the case for the harbor seal and fur seal data.

Unlike the Weibull curve, the Siler curve is designed to fit to three general stages of life—a juvenile stage with increasing survival, an adult stage with stable survival, and a late stage with decreasing survival. The Siler curve worked well for the fur seal and harbor seal data, although we did not fit it to the juvenile data because it resulted in a relatively poor fit to the adult ages (which were our primary focus). We did not fit the Siler model to the Steller sea lion data because it was necessary to keep the baseline vital rate data equivalent to the previously published Steller sea lion models for an equitable comparison across model types. The relative ease with which alternative curves can be selected illustrates that virtually any traditional mortality curve can be substituted for Eq. 2 to tailor the model to the biological interests of the investigator. In general we calculated survival rates from the numbers of animals-at-age reported in the original data sources, except in the case of the Steller sea lion, where we obtained age specific rates directly from Holmes et al. [5].

We diverted slightly from the Holmes et al. [5] method in interpreting the maturity data by assuming that individuals that were said to have ovulated or were pregnant at age 3 y were actually between 3 and 4 years of age, and would thus have given birth at age 4 y. We multiplied this probability by the survival from age 3 to 4 y to calculate the probability of a pregnant 3 year old giving birth at age 4 y. Holmes et al. [5] did a similar adjustment, but multiplied the pregnancy probabilities by the probability of survival from age 2 to 3 y, which is a minor difference in interpretation of the pregnancy data and probably would not affect the results in any meaningful way.

Juvenile survival rates were held constant over the age range appropriate for juvenile status for northern fur seals and harbor seals (transitions 0–1 y and 1–2 y). Following Holmes et al. [5], survival over the first 3 years for the Steller sea lions (0–1 y, 1–2 y and 2–3 y) increased in value.

*Step 2—multiplying survival and maturity curves and fitting to fertility data.* We multiplied Eqs. 1 and 2b or 2c (depending upon the species) together to give a final age-based fertility (*F*), where fertility is defined as the average number of pups born to a female of age *x* per year. It is important to note that we assume pregnancies result in a single offspring as is the case in our example species.
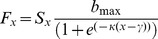
(3a)We then adjusted the logistic parameter that defines the asymptote *b_max_* to minimize the sum of squares between the multiplied curves (Eq. 3a) and late term age specific pregnancy data. We limited this multiplication to those ages (*x*) where pregnancy in the *data* is non-zero which does two things. The first is that survival at this age is very close to or at its peak, so that we are primarily using *S_x_* as a proxy for senescence. Second, it prevents creating births, near the tail of the curve at young ages, that do not occur in nature.

*Step 3—filling in the Leslie matrices.* The *S_x_* terms can be directly inserted into the subdiagonal of the Leslie matrix, however, because our matrices were birth pulse, the *F_x_* needed to be multiplied by the survival term *S_x_* for a final fertility term [6]

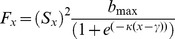
(3b)


All of the models (Leslie matrices) for the three species of pinnipeds were initially non-stationary, producing either a slightly increasing or decreasing population over time. However, the data used to create the matrices were assumed to come from a stationary population. We therefore multiplied all of the vital rates in the Leslie matrix by a common adjustment factor to obtain initial stationary populations as per Holmes et al. [5]. These types of adjustments are common to force stationarity in the transition matrix when transverse data collection methods are used, as is the case in all the example data sets we used here. This undoubtedly has implications for the interpretation of results though there is little information in the literature on the magnitude of influence it could have. Under such circumstances, we advise caution when interpreting the results. Given longitudinally collected data sets where stationarity is not a necessary assumption to be met, out methods here still apply.

**Figure 1:**
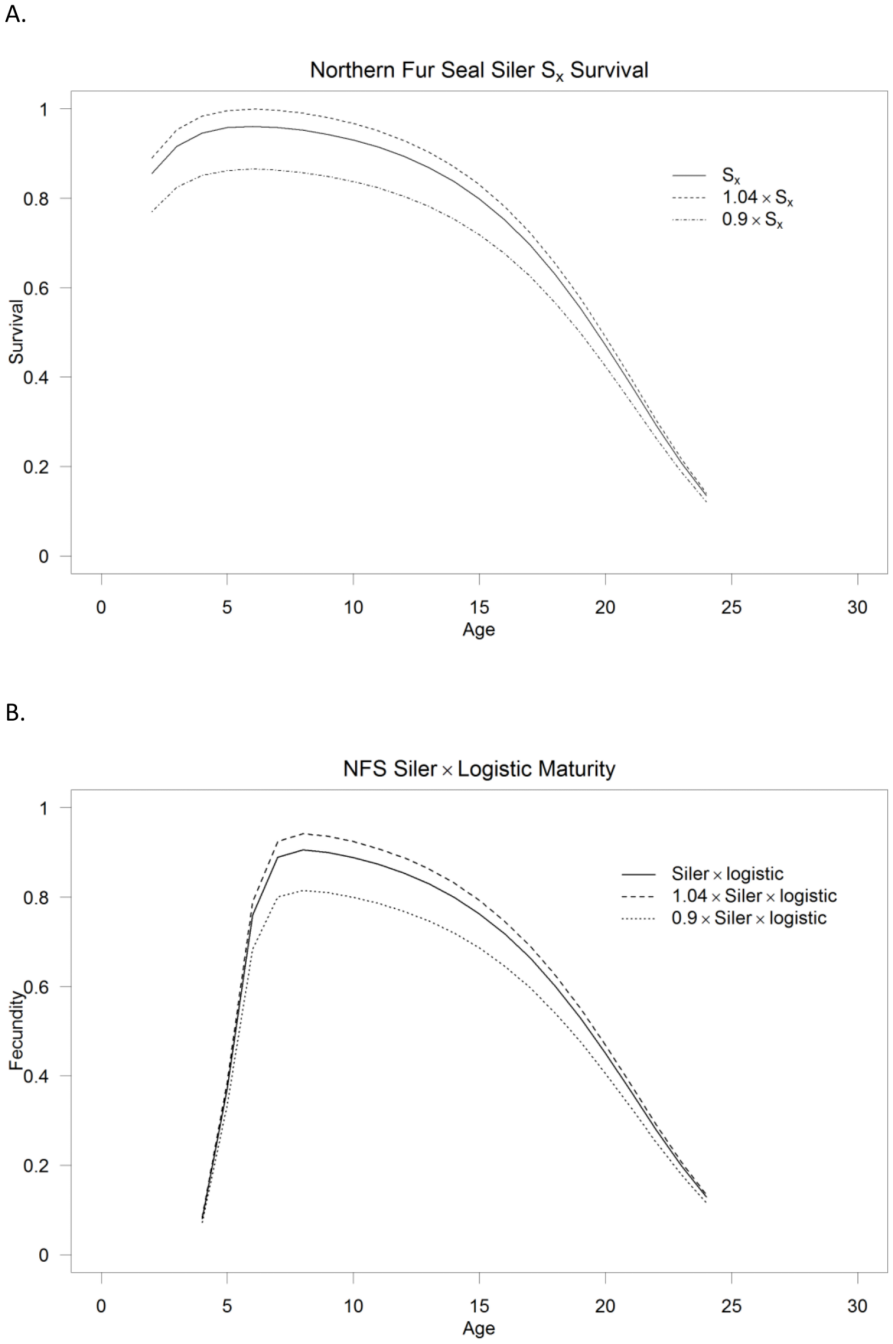
Vital rate adjustment. Examples of how northern fur seal survival (*S_x_*) (Fig. A) and fertility (*F_x_*) (Fig. B) changes over age when multiplied by a constant for fitting the models to count data by adjusting the vital rates in Leslie matrices that best describe the population dynamics over different time periods.

*Step 4—fitting models to count data.* We generally followed the model fitting techniques of Holmes et al. [5] where they adjusted vital rates of the Leslie matrix over different time period blocks within the range of the population count data. The version of Eq. 3a that we used for the Steller sea lions is the easiest to understand as the Weibull has a parameter that acts as an intercept (*m_0_*). So, replacing *S_x_* in Eq. 3a with Eq. 2c results in
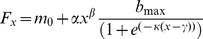
Hence, simply increasing or decreasing *m_0_* increases or decreases both *F_x_* and *S_x_* simultaneously. Using Eq. 2b (a function of the Siler curve-2a, which does not have a scalar or intercept type parameter) instead of Eq. 2c for replacement of *S_x_* in Eq. 3 means that the entire equation (Eq. 2b) can simply be multiplied by an adjustment factor, or an adjustment factor can be added depending upon how one might want to adjust the curves. Adding an adjustment factor will act like an intercept moving the curve up or down without adjusting its relative shape—while a multiplier will both move the curve up and down and result in larger survival or fertility values (early adult) being affected in greater absolute terms than ages with lower survival or fertility (essentially stretching or compressing the curve as if it were anchored at 0, for both *S_x_* [Fig. 1a] and *F_x_* [Fig. 1b]).

Linking Eqs. 1 and 2 together means that only one parameter needs to be estimated to adjust both the survival and fertility when fitting the Leslie matrices to the population count data. Not formulating Eq. 3a in this manner would mean that the fertility and survival vital rates would need to be independently adjusted. Reducing the number of parameters in this way is the major advantage of the RSSL model when determining the most parsimonious model. Other estimated parameters included the adjustment to juvenile survival, the starting number of pups to initialize the population, and variance parameters (one for each type of observed data). The Steller sea lion data also had two additional parameters (*p_2_* and *p_3_*) to correct for the difference between observed animals and the number of actual animals (details can be found in [5]). Table 1 indicates the number of and specific parameters that were estimated for each model by species.

**Table 1:** Numbers of estimated parameters for the fits to population data.

	RSSL w J/T	HFYS w J/T	RSSL	HFYS	FS HS RSSL	FS HS Stnd
Estimated Parameters						
σ ln Pups	1	1	1	1	1	1
σ ln Non-Pups	1	1	1	1		
σ ln Juvenile/Total	1	1				
Survival juvenile	4	4	4	4	3	3
Survival adult		4		4		3
Fertility		4		4		3
Adult survival curve scalar	4		4		3	
Initial number of pups	1	1	1	1	1	1
p_2_	1	1	1	1		
p_3_	1	1	1	1		
Total	14	18	13	17	8	11

RSSL is the Reproductive and Somatic Senescence Linked model, HFYS is Holmes et al. [5], NFS is northern fur seals (*Callorhinus ursinus* (L., 1758)), HS is harbor seals(*Phoca vitulina richardii* (Gray., 1864)), Stnd is the standard models from the literature, J/T is the proportion of juveniles to total non-pups data, *σ* is the variance of the data and *p_2_* and *p_3_* are parameters in the Holmes et al. [5] model that relate the number of animals visually detected in counts to the actual numbers in the population.

In fitting the Steller sea lions models, we used the negative log-likelihood function of Holmes et al. ([5] their Eq. 7), which included minimizing the difference between pup counts, adult counts and a juvenile to total-non-pups ratio, to model estimates. Our negative log-likelihood function for fur seals utilized only the pup counts while our harbor seal function utilized only the adult counts portion of the Steller sea lion likelihood. The likelihood equation for pup counts and adults counts was the same and is
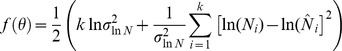
where θ is our set of estimated parameters (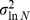, 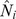), *k* is the number of observations and 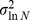 is the variance of the data calculated as
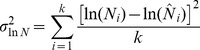
where 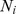 is the number of animals observed in year *i* and 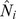 is the model estimated number of animals.

We compared different models using AIC corrected for a small sample using AIC_c_ = −2log(L(*θ*))+2*K*+2*K*(*K*+1)/(*n*-*K*-1) (p.66, [41]), where L(*θ*) is the likelihood, *K* is the number of estimated parameters and *n* is the sample size. For the Steller sea lions, we compared the RSSL model to the Holmes et al. [5] model as well as the Winship and Trites [42] model, and the Calkins and Pitcher [32] model as formulated in Holmes et al. [5]. An unadjusted non-stationary RSSL model for Steller sea lions grew at approximately 2.2% per year which we compared to a Holmes et al. [5] model that increased by 0.4% each year. This was the annual rate (0.4%) that the York [7] model (the ancestor of the York and Holmes family of Steller models) increased prior to being made stationary by decreasing the juvenile survival rate. Hence, for the Holmes et al. [5] model, we adjusted the age 0–1 survival rate, and linearly interpolated the age 1–2 and 2–3 y survival rates between the ages of 1 and 4 y so that the final growth rate would be 0.4%. This was essentially a reverse engineering of the method used by Holmes et al. [5] to form their stationary matrix.

### Species specific estimation of survival and fertility rates

#### Fur seals

*Step 1.* Female survival rates came from Table 7 of Lander [31], and female pregnancy rates came from Table 4 of Lander [31]. We fit the Siler curve to the female survival data from ages 3 to 25 y, and fit the logistic curve to the first 12 years of pregnancy rate data after which the pregnancy rate began to drop off. We used pregnancy data as a proxy for maturity because ovulation based maturity data were not available. Hence, we only used the first 12 years to fit the logistic curve because pregnancy rates declined with age and an individual that became sexually mature could not subsequently become immature.

**Figure 2:**
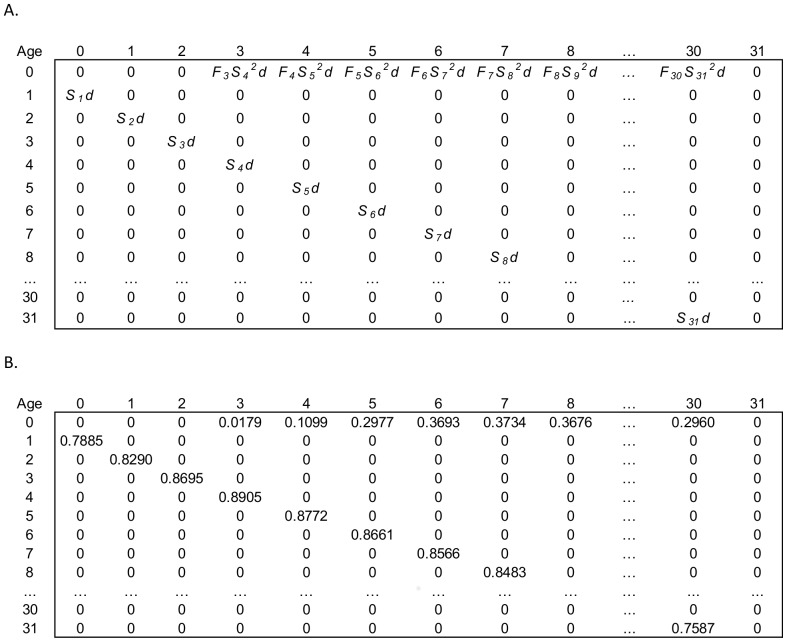
Final transition matrices for the Reproduction and Somatic Survival Linked (RSSL) model. A. The multiplication of Fertility *F* and Survival *S* values from Table 1 and the initial stationary population adjustment parameter *d*. B. Final RSSL Leslie matrix for Steller sea lions (*Eumetopias jubatus* (Schreber, 1776)).

*Step 2.* We multiplied the logistic and survival curves together (using ages 4 to 25) and adjusted the *b_max_* term of the logistic equation to fit Eq. 3 to the pregnancy data (Table 4, [31]) using least squares.

*Step 3.* The fertility transition terms for the Leslie matrix were multiplied by 0.5 to determine female only births, which were again multiplied by the age specific survival rate. Juvenile survival (0–1 y and 1–2 y) was set to the square root of 0.4 (taken from [31]). These give us the final vital rate terms for the Leslie matrix which were multiplied by the adjustment factor *d* to achieve an initial stationary population.

#### Steller sea lions

*Step 1.* The raw data for the Steller sea lions came from Calkins and Pitcher [32], and has been analyzed extensively by many others. We used survival rates from ages 3 to 30 y based on a version of the Weibull model from York [7] to fit to a parameterization of the Weibull from Ricklefs and Scheuerlein [40] (Eq. 2). We realized that fitting a Weibull curve to data generated from fitting an alternate parameterization of a Weibull curve to raw data was contrived. However, the original data that York [7] used was not available and we preferred the Ricklefs and Scheuerlein [40] parameterization as it is more intuitive biologically for our purposes. We fit a logistic model (forced to an asymptote of 1) to the ovulation data from Table 17 of Calkins and Pitcher [32].

*Step 2.* We obtained a close fit to the pregnancy data (Table 7 of [32]) after multiplying the Weibull survival and maturity curves (using ages 3–30). No adjustment to the intercept of the maturity curve was required to fit the fecundity RSSL curve to the pregnancy data that did not include the last 10 years of data. We did not include the last 10 years of data because they were based on a sample size of only 3 animals and because those 10 years represented a relatively small portion of the population (i.e., <2%). We thus assumed that extending the RSSL curve to age 30 y represented the reproductive status of females from age 20 to 30 better than the reproductive status that the 3 sampled individuals indicated.

*Step 3.* Juvenile survival 0–1, 1–2 and 2–3 y came directly from Holmes et al. [5]. The fertility transition terms for the Leslie matrix were multiplied by 0.5 to determine female only births, which were again multiplied by the age specific survival rate. This yielded the final vital rate terms for the Leslie matrix which we multiplied by the adjustment factor *d* to achieve an initial stationary population (see Table 2 for the fertility and survival values prior to adjusting for an initial stationary population and Fig. 2 for the algorithm for formulating the final Leslie matrix).

**Table 2:** Steller sea lion vital rates.

	RSSL		Calkins Pitcher 1982		Winship Trites 2006		Holmes et al. 2007	
Age	Survival	Fertility	Survival	Fertility	Survival	Fertility	Survival	Fertility
0		0		0		0		0
1	0.8060	0	0.7625	0	0.8001	0	0.8060	0
2	0.8474	0	0.7977	0	0.8334	0	0.8474	0
3	0.8888	0.0197	0.8328	0	0.8667	0	0.8888	0
4	0.9103	0.1234	0.8680	0.1008	0.9000	0	0.9302	0.0480
5	0.8967	0.3394	0.8790	0.1796	0.9000	0	0.9092	0.1695
6	0.8854	0.4263	0.8880	0.2615	0.9000	0.3150	0.8951	0.2215
7	0.8757	0.4358	0.8930	0.3150	0.9000	0.3150	0.8839	0.2795
8	0.8672	0.4334	0.8980	0.3150	0.9000	0.3150	0.8746	0.3285
9	0.8596	0.4298	0.8740	0.3150	0.9000	0.3150	0.8665	0.3285
10	0.8528	0.4264	0.8990	0.3150	0.9000	0.3150	0.8593	0.3285
11	0.8466	0.4233	0.8930	0.3150	0.9000	0.3150	0.8528	0.3850
12	0.8408	0.4204	0.8960	0.3150	0.9000	0.3150	0.8468	0.3850
13	0.8355	0.4178	0.8950	0.3150	0.9000	0.3150	0.8412	0.3850
14	0.8306	0.4153	0.8950	0.3150	0.9000	0.3150	0.8360	0.3850
15	0.8259	0.4130	0.8950	0.3150	0.9000	0.3150	0.8312	0.3850
16	0.8216	0.4108	0.8950	0.3150	0.9000	0.3150	0.8266	0.3850
17	0.8175	0.4087	0.8950	0.3150	0.9000	0.3150	0.8223	0.2570
18	0.8136	0.4068	0.8950	0.3150	0.9000	0.3150	0.8182	0.2570
19	0.8099	0.4049	0.8950	0.3150	0.9000	0.3150	0.8142	0.2570
20	0.8063	0.4032	0.8950	0.3150	0	0.3150	0.8105	0.2570
21	0.8030	0.4015	0.8950	0.3150	0	0	0.8069	0.2570
22	0.7997	0.3999	0.8950	0.3150	0	0	0.8034	0
23	0.7967	0.3983	0.8950	0.3150	0	0	0.8001	0
24	0.7937	0.3968	0.8950	0.3150	0	0	0.7968	0
25	0.7908	0.3954	0.8950	0.3150	0	0	0.7937	0
26	0.7881	0.3940	0.8950	0.3150	0	0	0.7907	0
27	0.7854	0.3927	0.8950	0.3150	0	0	0.7878	0
28	0.7828	0.3914	0.8950	0.3150	0	0	0.7850	0
29	0.7803	0.3902	0.8950	0.3150	0	0	0.7822	0
30	0.7779	0.3890	0.8950	0.3150	0	0	0.7795	0
31	0.7756	0.3878	0.8950	0	0	0	0.7769	0

Survival and fertility vital rates by age of four Steller sea lion matrices. Vital rates are prior to adjustment for a stationary population and the fertility rates are prior to multiplication by survival rates to fill in the matrix fertility transitions. RSSL is the Reproduction and Somatic Survival Linked model.

#### Harbor seals

*Step 1.* We obtained the female survival rates from Table 2 of Pitcher [2], and estimated the adult survival rates using data from age 0 to 31 y by fitting a Siler survival curve. We were unable to ignore the males in the harbor seal population because the adult count data included both males and females. We estimated male survival rates using data from age 0 to 27 y by again fitting a Siler survival curve. We fit the logistic curve to ovulation rates from Table 6 of Pitcher and Calkins [22] and forced it to have an asymptote of 1 (100% maturity).

*Step 2.* We multiplied the logistic maturity and Siler survival curves together (using ages 4–31) and adjusted the *b_max_* term of the logistic equation to fit Eq. 3a to the pregnancy data from Table 6 of Pitcher and Calkins [22] using least squares.

*Step 3.* Female and male juvenile survival came directly from Table 2 of Pitcher [2]. The fertility transition terms for the Leslie matrix were multiplied by 0.5 to determine female only births, which were again multiplied by the age specific survival rate. These give us the final vital rate terms for the Leslie matrix which were multiplied by the adjustment factor *d* to achieve an initial stationary population.

### Leslie Matrix model fitting to time series counts (Step 4)

#### Equations

We calculated the age specific number of fur seals and harbour seals using the estimated demographic parameters (fertilities and adult and juvenile survival rates) and the following equations:
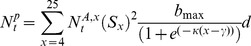
(4)

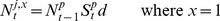
(5a)

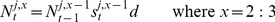
(5b)

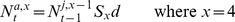
(6a)

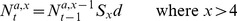
(6b)and *N_t_* is the number of pups (*p*), juveniles (*j*) or adults (*a*) of age *x* in year *t*, and *d* is the adjustment for the initial stationary population. In matrix notation, these equations simplify to
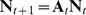
where **N** is the vector of numbers of animals and **A** is the Leslie transition matrix at time *t*. Eqs. 4–6 were equivalent for the Steller sea lions, with the exception that *S_x_* is Eq. 2c (Weibull curve) instead of Eq. 2a–b (Siler curve). Again, the reduction in parameters for the RSSL occurred because *S_x_* occurred in both Eqs. 4 and 6.

#### Population Count Data

**Figure 3:**
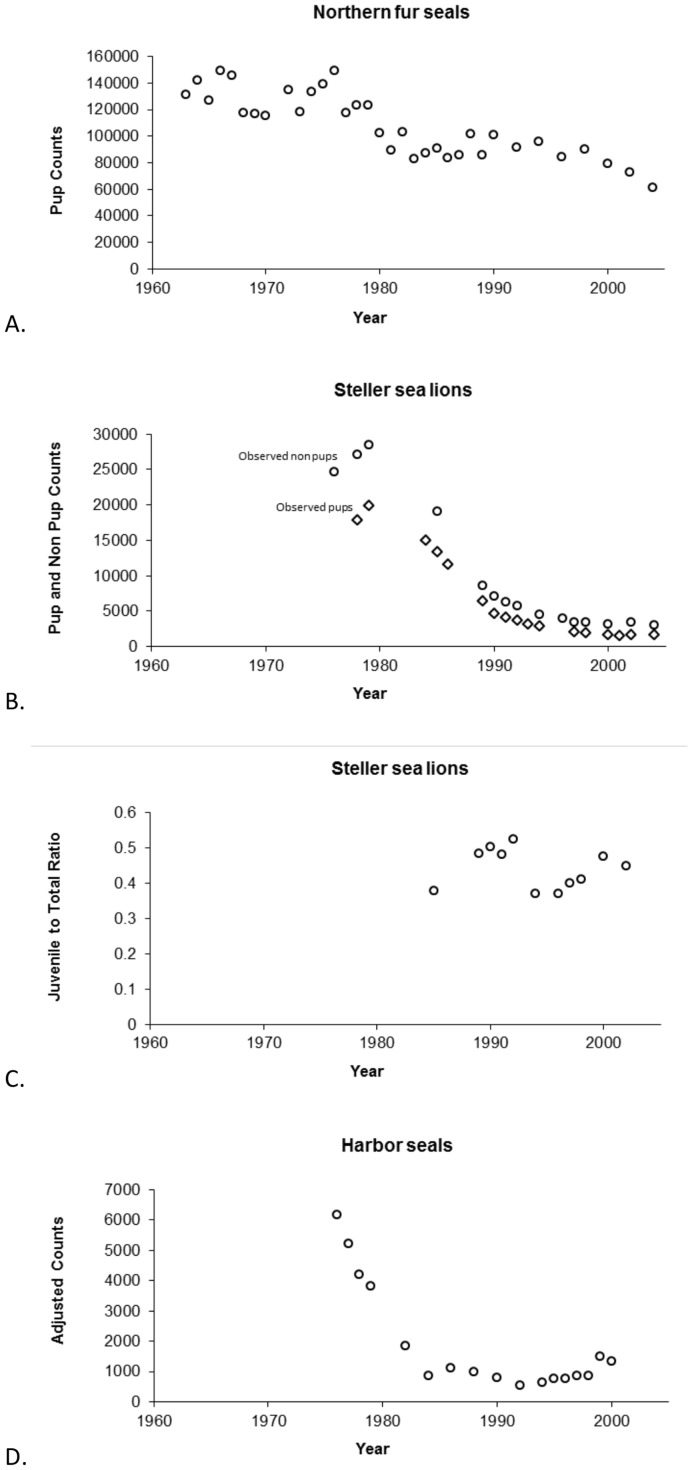
Time series counts for north pacific pinnipeds. A. Northern fur seals (*Callorhinus ursinus* (L., 1758)) at St. Paul Island, Alaska, from the National Marine Fisheries Service Annual Fur Seal Investigation Reports and Lander [43], (B&C) Steller sea lions (*Eumetopias jubatus* (Schreber, 1776)) in the Central Gulf of Alaska from Holmes et al. [5] and (D) harbor seals (*Phoca vitulina richardii* (Gray., 1864)) at Tugidak Island, Alaska, from Jemison et al. [44].

Fur seal data (Fig. 3a) came from either Lander [43] for the early counts or the Fur Seal Investigation Reports that were published annually by the US National Marine Fisheries Service. The best available population abundance data were pup counts that were estimated using mark recapture techniques and have been made annually or biannually for the last half of the 1900's. Unfortunately, while an incredible amount of data on northern fur seals has been gathered over the last century; absolute counts or indexes on the number of females do not exist, making it difficult to determine the percentage of the population that returns each year or is not on land at any one time.

Steller sea lion data (Fig. 3b–c) came from aerial surveys of the major rookeries and haulouts from the central Gulf of Alaska [5]. Pup counts were from the five major rookeries (Marmot, Sugarloaf, Chowiet, Chirikof, and Outer Islands), while non-pup counts came from all rookeries and haulouts designated as “trend” sites, and the juvenile to total non-pups ratio came from all haul-outs photographed in aerial surveys from 1985 to 2002.

Harbor seal data (Fig. 3d) came from Jemison et al. [44] and were collected by counting animals using spotting scopes from cliffs above where the animals had hauled out on the beaches during the peak pupping and molting periods from May to September. These data were subsequently adjusted by accounting for environmental factors such as tide height, time of day and weather conditions, among others, as performed by Jemison et al. [44].

#### Adjusting vital rates

We chose to adjust the vital rates three or four times for each species over the time period with which we had data. For the Steller sea lions, we used the same years (1984, 1989, 1993 and 1998) as were found for the most parsimonious model in Holmes et al. [5]. Dates for the fur seal and harbor seal were determined by eyeballing inflection points in the trends of the data. We were aware that “eyeballing” was not a valid technique for determining parsimony, and in applications for biological inference, numerous models would need to be run to determine the most parsimonious numbers and locations of inflection points. In this case, however, we were only interested in comparing model structures and comparing all of them under the same conditions. As such, eyeballing inflection points was unlikely to make a difference in this instance so long as the conditions were biologically reasonable. For the fur seals, we used 1975, 1985, and 1995; while for the harbor seals we used 1977, 1985 and 1993. We used the native minimizing routine solver of MS Excel to optimize the maximum likelihood function and fit the Steller sea lion models twice (once to the pup and non-pup count data only and once including the juvenile to total non-pups ratio in addition to the pup and non-pup count data). Models for the northern fur seal and harbour seal were written in R [45] (see Appendix A for R code using the northern fur seal data as an example). Estimated parameters were constrained to ensure that the fertilities and juvenile and adult survival rates stayed within biologically realistic ranges.

Unlike the Steller sea lions which required the additional parameter *p_2_* and *p_3_* parameters to adjust for uncounted animals in the data, we did not need to adjust the number of harbor seals counted to those actually present because these calculations had already been done by Jemison et al. [44] unlike the Steller sea lion models. In addition, we considered the pup counts for fur seals to be estimates of the absolute number and not an index—so no assumptions were required to adjust to actual numbers.

Because the harbour seal count data included both sexes, we adjusted the rates for both the juvenile and adult male survival by the same amount as the corresponding female rates, meaning that no additional parameters were estimated to fit the models to the count data. For example, if the female adult survival rate decreased by 10%, we reduced the adult male survival rate by 10%.

## Results

### Species specific estimation of survival, maturity and fertility rates

**Figure 4:**
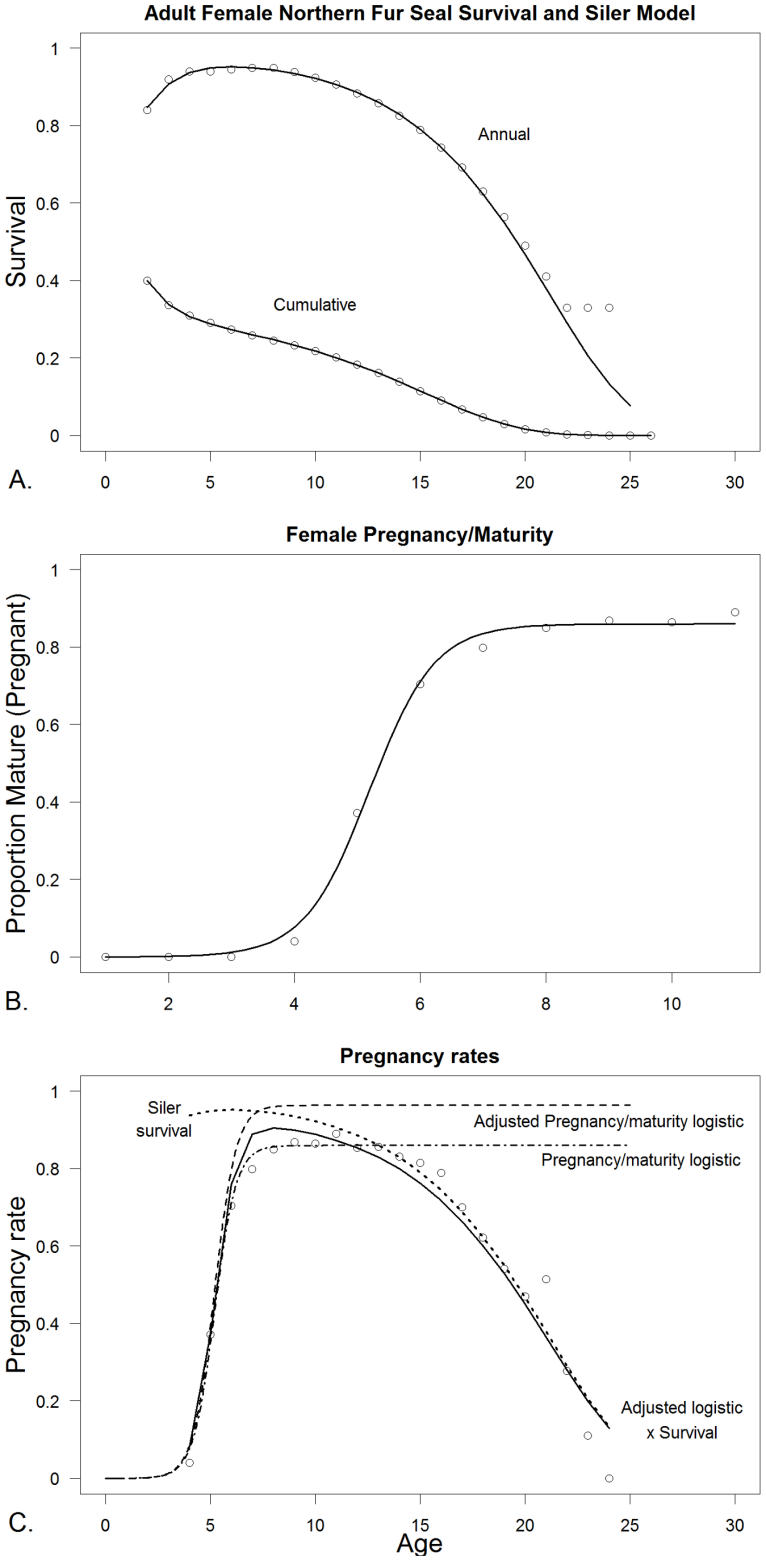
Estimation of survival, maturity and pregnancy rates for Northern fur seals (*Callorhinus ursinus* (L., 1758)). A. Adult female survival data from age 4 to 25 with the fitted Siler curve (Eq. 2a). B. Female maturity rate based on late term pregnancy data from the first 11 age classes with the fitted logistic curve (Eq. 1). C. Female pregnancy rates from fitting the multiplication of the survival curve and the maturity curve extended out to age 25, by adjusting the scalar of the logistic maturity function, in this case up (Eq. 3). All data are from Lander [31].

**Figure 5:**
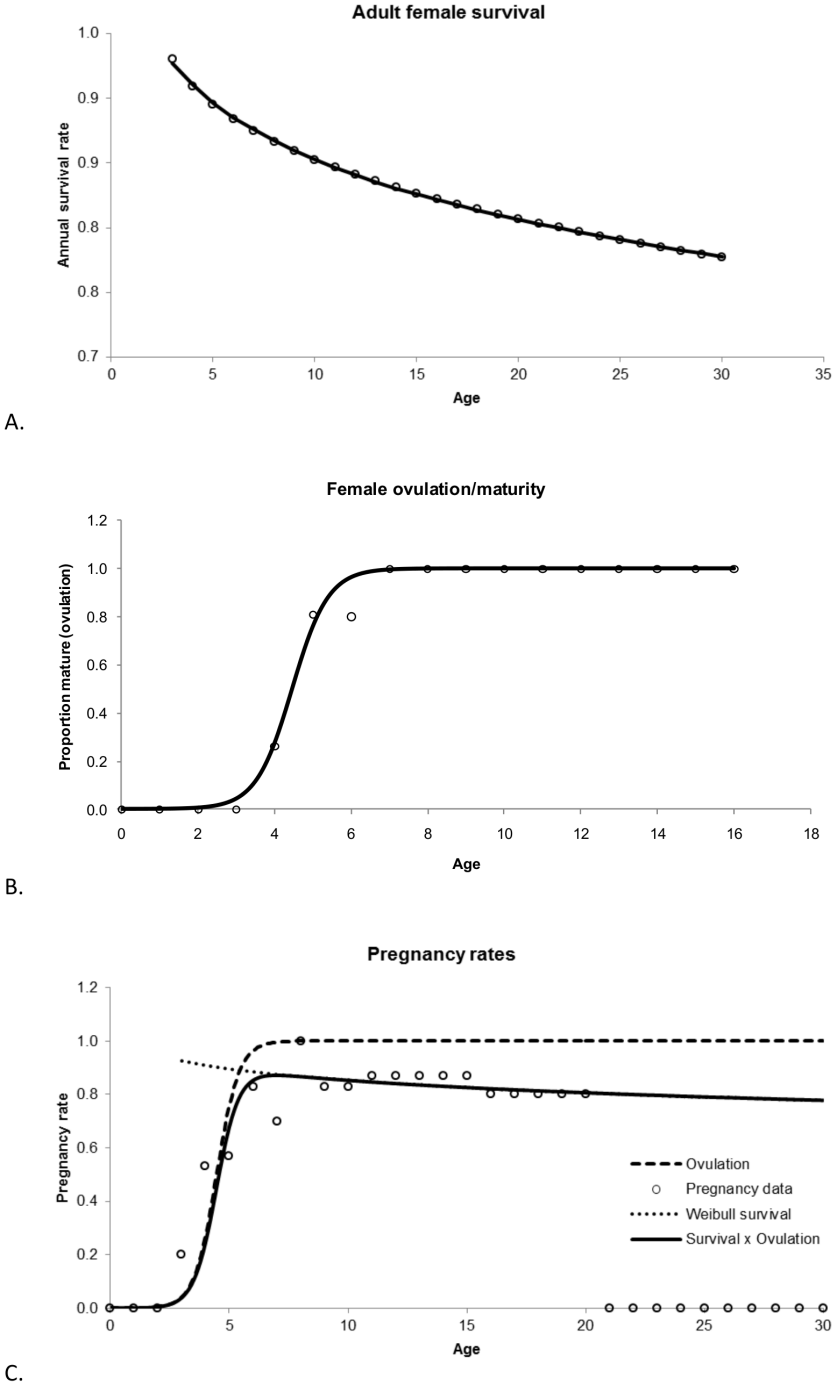
Estimation of survival, maturity and pregnancy rates for Steller sea lions (*Eumetopias jubatus* (Schreber, 1776)). A. Adult female survival data from the York [7] Weibull model, using age 3 to 30 y with the fitted Weibull curve (Eq. 2c). B. Female maturity rate based on ovulation data with the fitted logistic curve (Eq. 1). C. Female pregnancy rates from the result of fitting the multiplication of the survival curve and the maturity curve by adjusting the scalar of the logistic maturity function, here, only late term pregnancy data to age 20 was used to fit the model to the data, as the data from age 21 to 30 y is represented by only 3 individuals.

**Figure 6:**
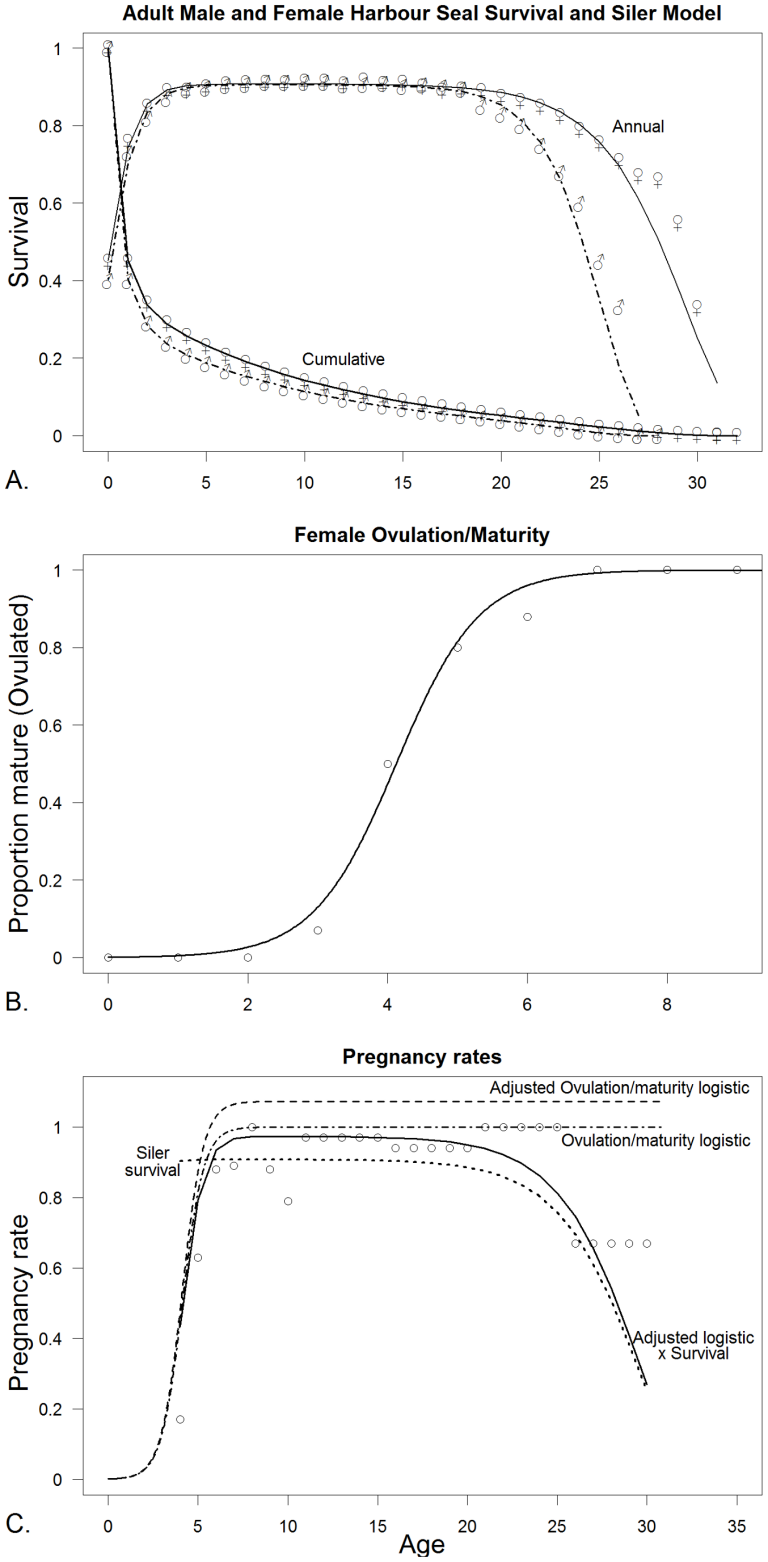
Estimation of survival, maturity and pregnancy rates for Harbor seals (*Phoca vitulina richardii* (Gray., 1864)). A. Survival data for both sexes and all ages showing the fitted polynomial for females age 4 to 27(Eq. 2a). Male survival rates were close enough to females from age 4 to 17 y that we used the female survival curve for males of those ages. B. Female maturity rate based ovulation data from ages 1–10 first age classes with the fitted logistic curve (Eq. 1). C. Female pregnancy rates from the result of fitting the multiplication of the survival curve and the maturity curve extended out to age 27 y, by adjusting the scalar of the logistic maturity function, in this case up. All data are from Pitcher [2], [22].

Table 2 indicates the number of and specific parameters that were estimated for each model. Table 3 shows the parameter estimates for the three species for the fitted survival and maturity curves as well as the stationarity adjustment to the Leslie matrix (*d*) and the adjustment to *b_max_* to fit the multiplication of the survival and maturity curves to pregnancy data. Figs. 4a and b show the Siler (R^2^ = 0.99) and logistic maturity curves (R^2^ = 0.99) fit to northern fur seals, while Fig. 4c shows the fit of the multiplication of the two with the adjustment of *b_max_* to fit to the pregnancy data (R^2^ = 0.95). Figs. 5a and b show the fitted survival Weibull (R^2^ = 0.97, which is artificially high) and logistic maturity (R^2^ = 0.97) for Steller sea lions, while Fig. 5c shows the fit to the pregnancy data after multiplying the survival and maturity curves (R^2^ = 0.80). Juvenile Steller sea lion survival age 0–1, 1–2 and 2–3 y was 0.8060, 0.8474 and 0.8888 prior to adjustment for the initial stationary population. Figs. 6a, b and c show the fitted survival Siler (R^2^ = 0.99), logistic maturity (R^2^ = 0.99) and multiplication of the two to fit to the pregnancy data for harbor seals (R^2^ = 0.49). R^2^ values were calculated as 
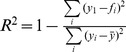
 where *y_i_* are observed values and *f_i_* are the fitted model values.

**Table 3:** Parameter summary for survival and maturity curves.

	Steller Sea Lion	Northern fur seal	Harbour Seal	
Siler			Female	Male
*a_1_*		0.8201	1.2153	1.3600
*b_1_*		0.7184	1.2423	1.1510
*a_2_*		0.0176	0.0963	0.0985
*a_3_*		0.0047	7.9930E-06	7.3280E-07
*b_3_*		0.2466	0.3928	0.5512
Weibull				
*alpha*	0.6535			
*beta*	0.0824			
*m_0_*	−0.6429			
Maturity logistic				
*kappa*	2.1252	1.9471	1.6926	
*gamma*	4.4652	5.1945	4.1229	
*b_max_*	0.5	0.8599 (0.9637)	1 (1.0728)	
Stationary Adjustment *d*	0.9783	1.0091	1.0080	

The adjustment to *b_max_* of the maturity logistic curve to fit the growth × maturity curve to pregnancy data (in parenthesis).

### Leslie matrix model fitting to time series counts

**Figure 7:**
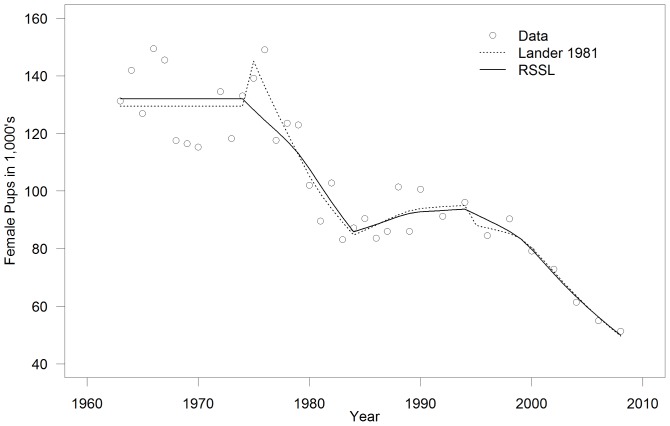
Northern fur seal (*Callorhinus ursinus* (L., 1758)) model fits. Reproduction and Somatic Survival Linked (RSSL) model and a model using data from Lander [31] fit to northern fur seal pup count data from St. Paul Island, Alaska. Both models have a relatively similar fit with the exception around 1975. Data and models are for the female portion of the population only.

**Figure 8:**
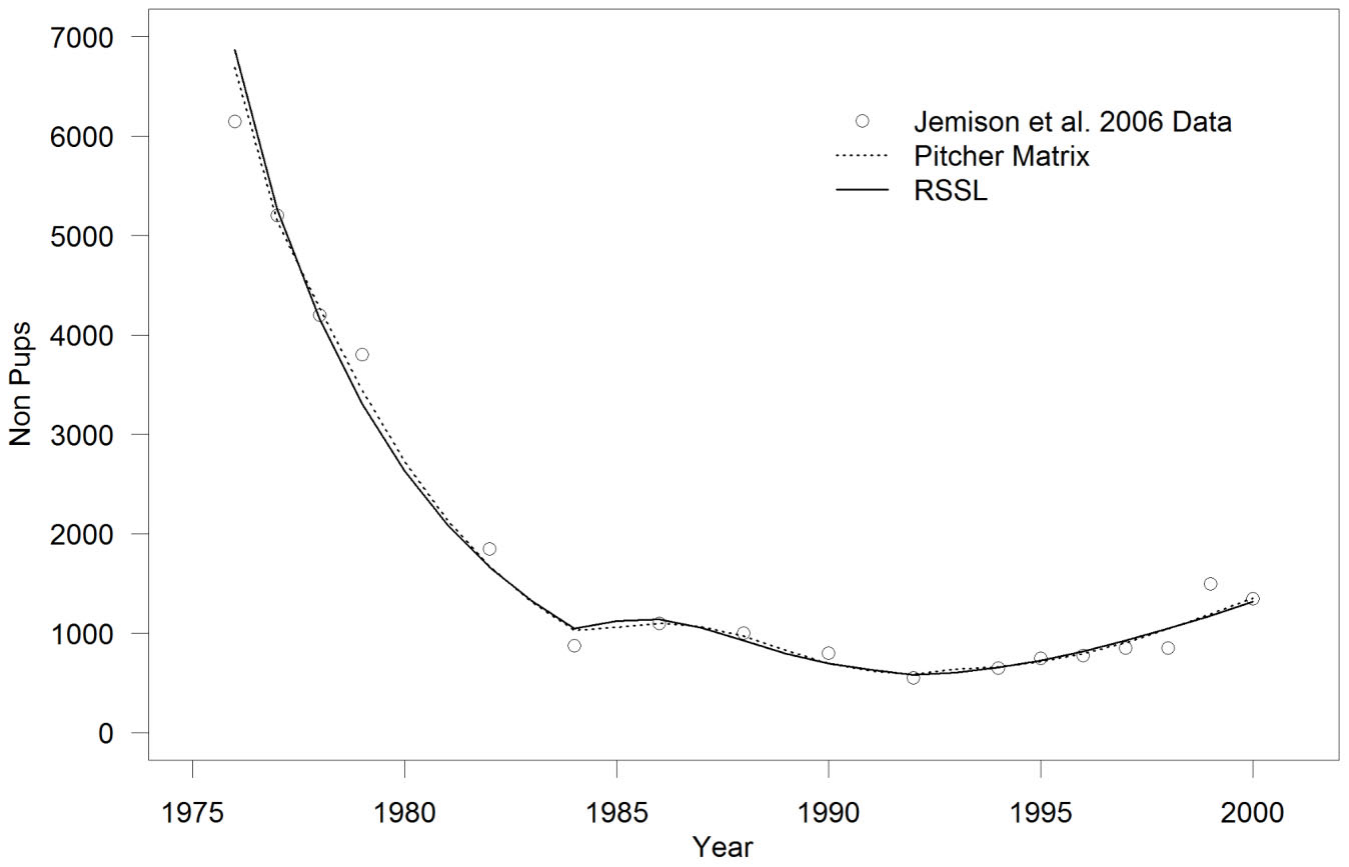
Harbor seal (*Phoca vitulina richardii* (Gray., 1864)) model fits. Reproduction and Somatic Survival Linked (RSSL) model and a model using data from Pitcher [2] fit to harbour seal count data from Tugidak Island, Alaska.

**Figure 9:**
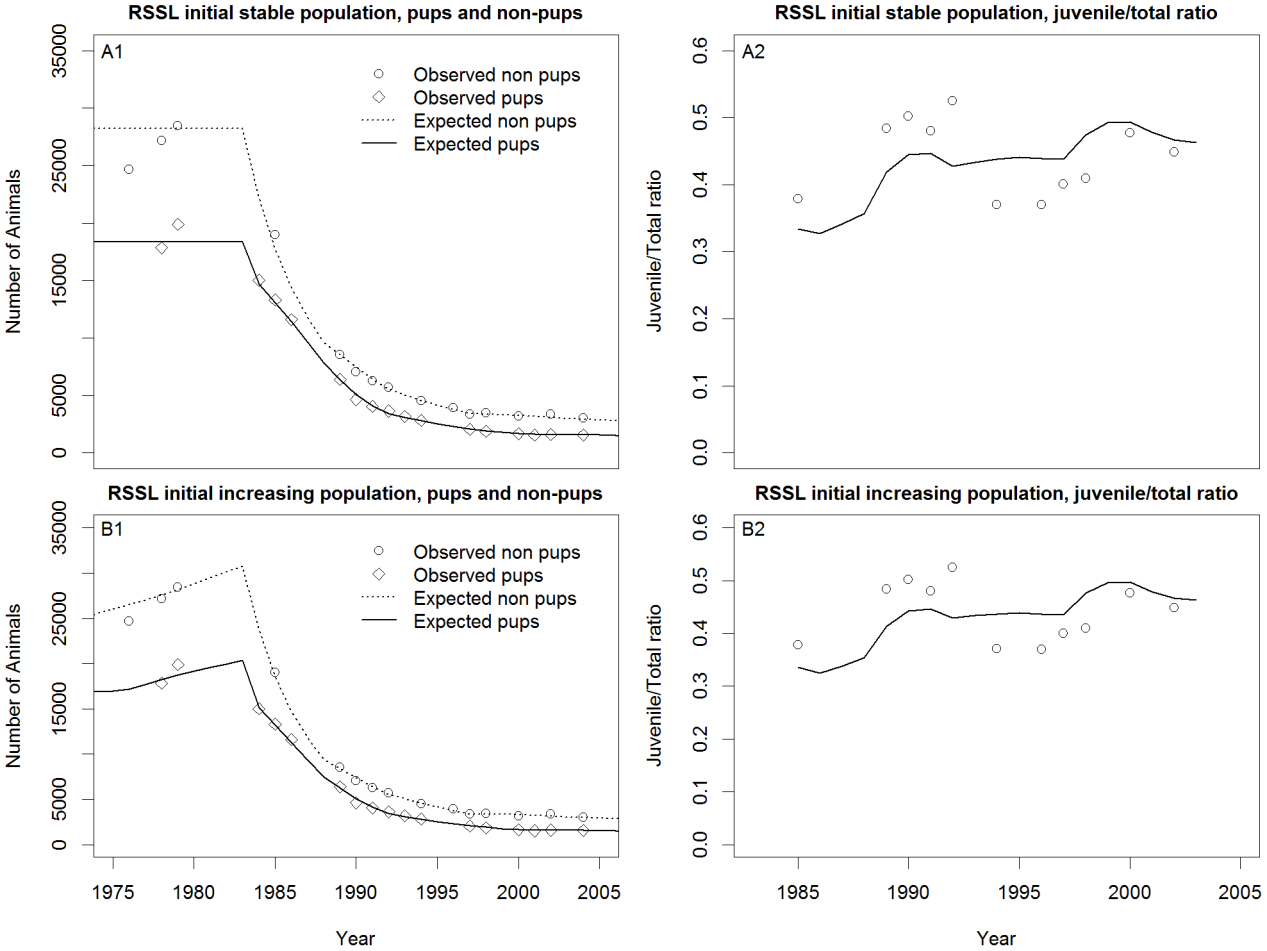
Steller sea lion (*Eumetopias jubatus* (Schreber, 1776)) model fits. Reproduction and Somatic Survival Linked (RSSL) initial stationary population (A1–A2) and RSSL initial increasing population (B1–B2).

**Figure 10:**
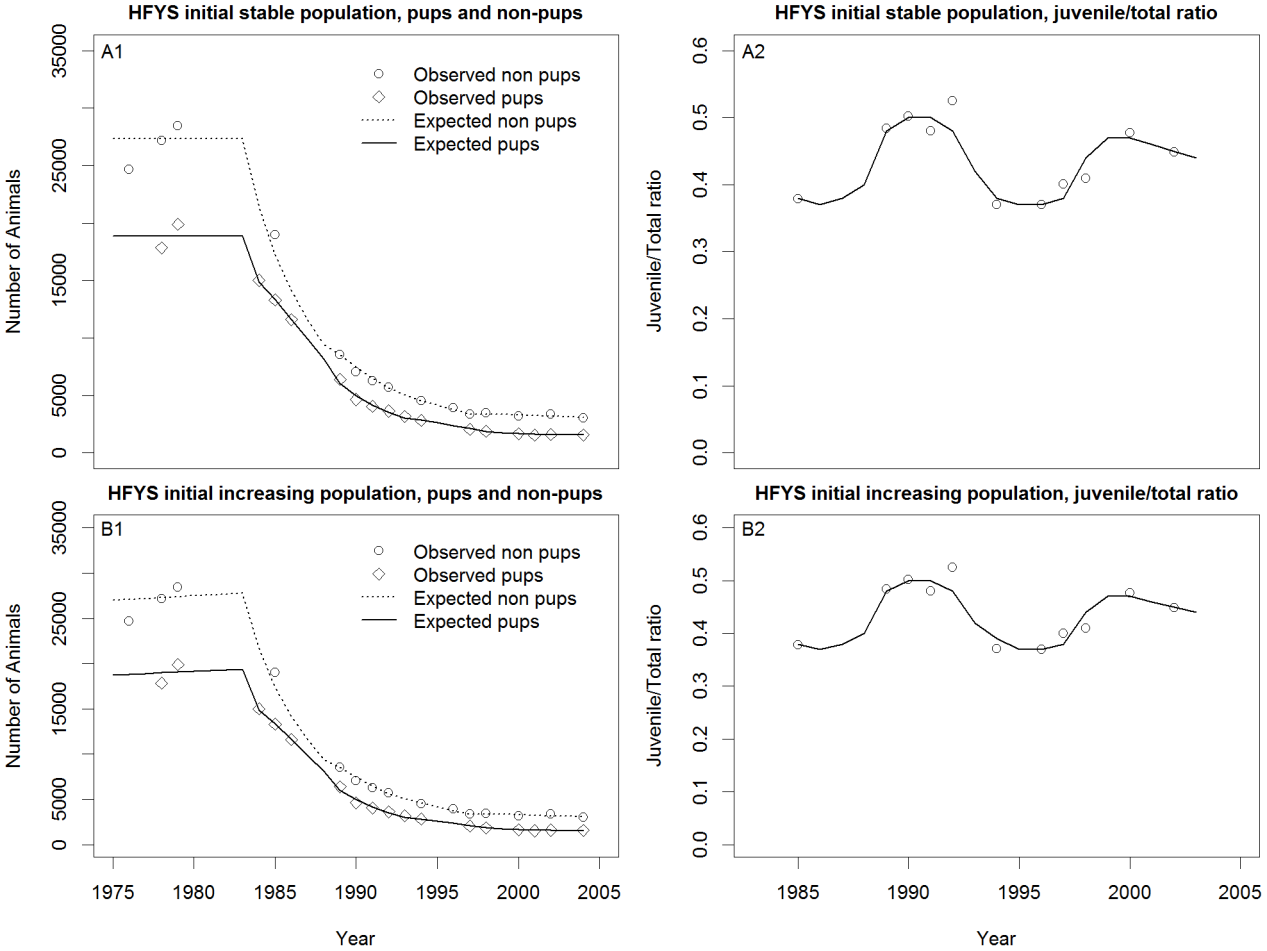
Steller sea lion (*Eumetopias jubatus* (Schreber, 1776)) model fits. Holmes et al. [5] (HFYS) initial stationary population (A1–A2) and Holmes et al. [5] initial increasing population (B1–B2).

**Figure 11:**
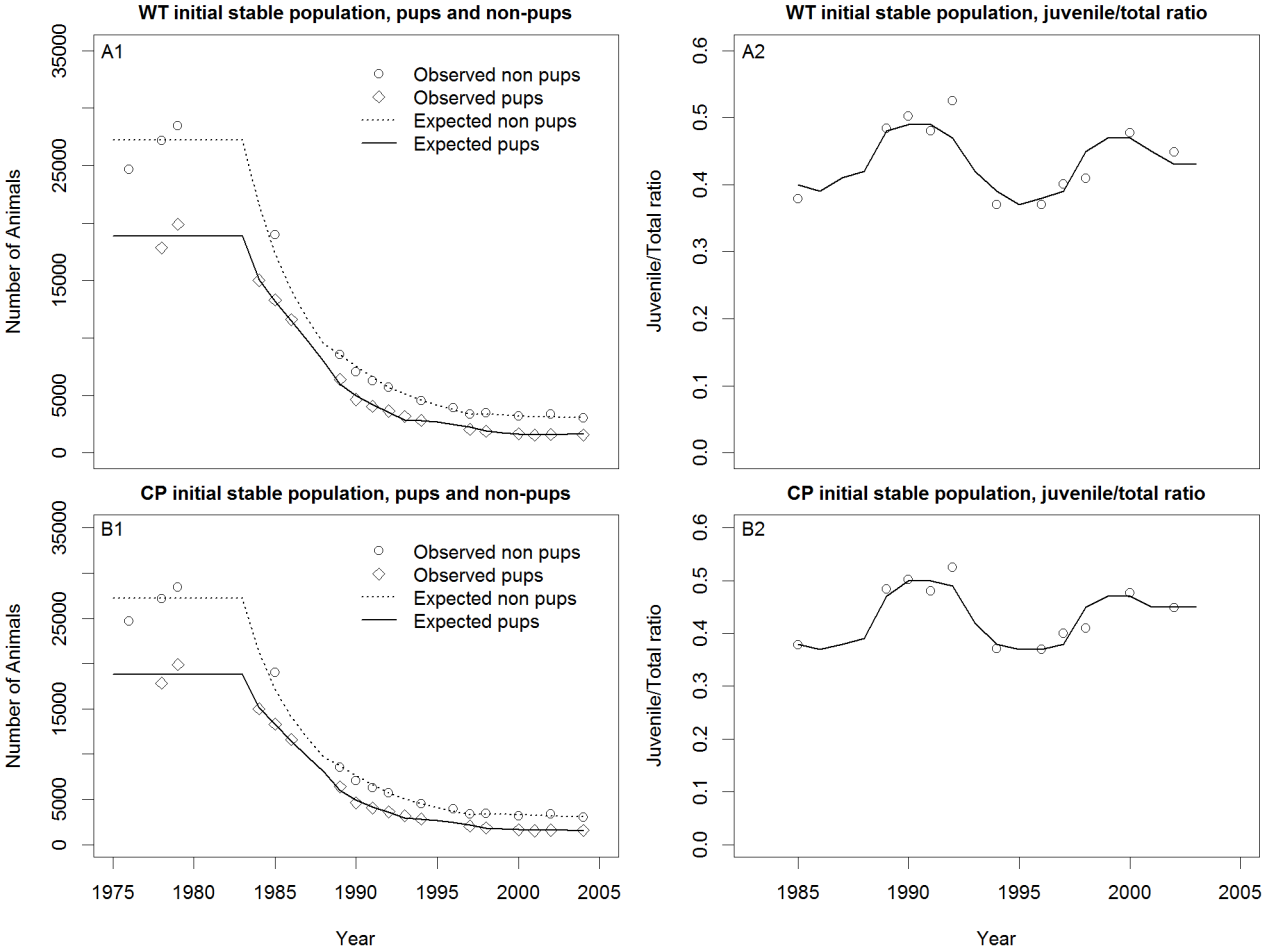
Steller sea lion (*Eumetopias jubatus* (Schreber, 1776)) model fits. Winship and Trites [42] (WT) (A1–A2) and Calkins and Pitcher [32] (CP) (B1–B2).

In all cases, the models visually fit the pup and non-pup counts quite well for all three species (Figs. 7–11) Pup and non-pup fits for the Steller sea lion models that did not use the juvenile to total non-pups ratio looked essentially the same as those for the models that did use the juvenile to total non-pups ratio. A summary of the results (Table 4) shows that the RSSL model outperformed the other models in all cases for the fur seal and harbor seals, as well as for the Steller sea lions when only using the pup and non-pup data to fit the models. However, the Holmes et al. [5] model performed better when using the juvenile to total non-pups ratio as was evident by the inability of the RSSL model to fit to the juvenile to total non-pups ratio data (Fig. 9). A representative sample of models showing the adjustments to birth and survival rates that resulted in the population trajectories in Figs. 7–11 are contained in Tables 5–7.

**Table 4:** Model AIC_c_ values.

MODEL	# Par	AIC_c_	Δ AIC_c_	# Data Points
**Fur Seal RSSL**	**8**	**−127.40**	**0**	**36**
Fur Seal Standard	11	−122.65	4.75	36
**Harbor Seal RSSL**	**8**	**−22.89**	**0**	**17**
Harbor Seal Standard	11	14.37	37.26	17
STELLER SEA LIONS				
RSSL w J/T	14	−177.40	5.81	43
**HFYS w J/T**	**18**	**−183.21**	**0**	**43**
Winship Trites w J/T	18	−169.11	14.10	43
Calkins Pitcher w J/T	18	−176.77	6.44	43
**RSSL**	**13**	**−121.32**	**0**	**32**
HFYS	17	−100.81	20.51	32
Winship Trites	17	−98.99	22.33	32
Calkins Pitcher	17	−97.95	23.37	32
**RSSL Increasing w J/T**	**14**	**−194.37**	**0**	**43**
HFYS Increasing w J/T	18	−185.41	8.96	43
**RSSL Increasing**	**13**	**−138.13**	**0**	**32**
HFYS Increasing	17	−89.91	48.22	32

HFYS is Holmes et al. [5], RSSL is Reproduction and Somatic Survival Linked, “w J/T” indicates that the Juvenile/(Juvenile+Adult) data was included in the Steller sea lion model and Standard indicates models created from unmodified Lander [31] or Pitcher [22] birth rates. The most parsimonious model based on AIC_c_ in each section is highlighted in bold.

**Table 5:** Survival, maturity and fertility parameter values for northern fur seals.

	Absolute change				% change multipliers		
Fur seal	Adult Survival Adjuster	Juvenile Survival Scalar	Maturity Scalar	Initial Pups 1000's	Adult Survival	Juvenile Survival	Fertility rates
Initial values	1.0000	0.6387	0.9235				
RSSL				*132.0 (2.912)*			
1975–1984	0.9713	0.5445	0.9235		*0.9713 (0.0098)*	*0.8525 (0.0841)*	0.9713
1985–1994	1.0408	0.5429	0.9235		*1.0408 (0.0316)*	*0.8500 (0.1544)*	1.0408
1995–2008	1.0167	0.4325	0.9235		*1.0167 (0.0459)*	*0.6771 (0.1635)*	1.0167
Lander							
1975–1984				*129.5 (2.7224)*	*0.9392 (0.0174)*	*0.9032 (0.0977)*	*1.1206 (0.0620)*
1985–1994					*1.0105 (0.0333)*	*0.8993 (0.1587)*	*1.2049 (0.1104)*
1995–2008					*0.9957 (0.0432)*	*0.6998 (0.0153)*	*1.1128 (0.1563)*

Adult and juvenile survival scalar, maturity scalar and fertilities derived from all the northern fur seal (*Callorhinus ursinus* (L., 1758) models. Italicized values are parameters that the models estimated. For the % change multipliers under the Reproduction and Survival Senescence Linked (RSSL) models, we used mean fertilities and adult survival over ages and time periods because these were non-linear functions of age and time. 95% confidence limits calculated via the hessian are in parenthesis. The non-RSSL models multiplied percentage change scalars directly with the Leslie matrix elements to affect changes over time while the RSSL changed *S_x_* which consequently changed the Leslie matrix elements.

**Table 6:** Survival, maturity and fertility parameter values for harbour seals.

	Absolute change					% change multipliers	
Harbor Seal	Adult Survival Adjuster	Juvenile Survival Scalar	Maturity Scalar	Initial Pups	Adult Survival	Juvenile Survival	Fertility rates
Initial values	1.0000	0.5848	1.0720				
RSSL				*1014(141)*			
1977–1984	0.8334	0.3299	1.0720		*0.8294 (0.2368)*	*0.5857 (1.3758)*	0.8334
1985–1992	0.7775	1.0000	1.0720		*0.7824 (0.1764)*	*1.7081 (1.3803)*	0.7775
1993–2005	1.1014	0.6911	1.0720		*1.1002 (0.1081)*	*1.1894 (0.5632)*	1.1014
Pitcher				*859 (60)*			
1977–1984					*0.7638 (0.8988)*	*0.7770 (0.0648)*	*1.0993 (0.5048)*
1985–1992					*0.5839 (0.2477)*	*1.3933 (0.2671)*	*1.0993 (0.8871)*
1993–2005					*1.0663 (0.3887)*	*1.3197 (0.4029)*	*0.8633 (1.0754)*

Adult and juvenile survival scalar, maturity scalar and fertility rates derived from all the harbor seal (*Phoca vitulina richardii* (Gray., 1864)) models. Italicized values are parameters that the models estimated. For the % change multipliers under the Reproduction and Survival Senescence Linked (RSSL) models, we used mean fertilities and adult survival over ages and time periods because these were non-linear functions of age and time. 95% confidence limits calculated via the hessian are in parenthesis. The non-RSSL models multiplied percentage change scalars directly with the Leslie matrix elements to affect changes over time while the RSSL changed *S_x_* which consequently changed the Leslie matrix elements.

**Table 7:** Survival, maturity and fertility parameter values for Steller sea lions.

	Absolute change				% change multipliers		
Steller Sea Lion	Adult Survival Scalar	Juvenile Survival Scalar	Maturity Scalar	Initial Pups	Adult Survival	Juvenile Survival	Fertility Rates
Initial values	−0.6429	0.8888	0.5000				
RSSL w J/T				*9679 (9269 10111)*			
1984–1988	*−0.5502 (−0.564 −0.536)*	*0.5475 (0.471 0.621)*	0.5000		0.8860	0.6135	0.8754
1989–1992	*−0.5569 (−0.580 −0.533)*	*0.7213 (0.659 0.783)*	0.5000		0.8866	0.8417	0.8784
1993–1997	*−0.5721 (−0.591 −0.552)*	*0.8168 (0.763 0.870)*	0.5000		0.9090	0.9095	0.8331
1998–2008	*−0.5803 (−0.593 −0.568)*	*0.9999 (0.977 LBR)*	0.5000		0.9325	1.1250	0.8079
RSSL				*9718 (9307 10147)*			
1984–1988	*−0.5492 (−0.563 −0.535)*	*0.5423 (0.476 0.613)*	0.5000		0.8873	0.6159	0.8777
1989–1992	*−0.5558 (−0.578 −0.534)*	*0.7481 (0.692 0.805)*	0.5000		0.8873	0.8116	0.8879
1993–1997	*−0.5680 (−0.586 −0.549)*	*0.8084 (0.755 0.861)*	0.5000		0.9128	0.9190	0.8433
1998–2008	*−0.5812 (−0.593 −0.570)*	*0.9999 (0.977 LBR)*	0.5000		0.9315	1.1250	0.8052
HFYS w J/T							
1984–1988					*0.8990*	*0.4200*	*0.8690*
1989–1992					*0.9280*	*0.7340*	*0.7620*
1993–1997					*1.0020*	*0.5650*	*0.7030*
1998–2008					*1.0680*	*0.9350*	*0.6410*
HFYS				*9920 (9440 10381)*			
1984–1988					*0.8947 (0.862 0.928)*	*0.6098 (0.471 0.730)*	*0.8908 (0.816 0.980)*
1989–1992					*0.9161 (0.875 0.960)*	*0.7871 (0.654 0.888)*	*0.7951 (0.683 0.927)*
1993–1997					*0.9667 (0.921 1.038)*	*0.8471 (0.642 0.949)*	*0.7882 (0.666 0.915)*
1998–2008					*0.9776 (0.945 LBR)*	*1.125 (0.930 LBR)*	*0.7695 (0.637 0.876)*
Calkins Pitcher w J/T				*9924 (9370 10512)*			
1984–1988					*0.8952 (0.846 0.937)*	*0.5456 (0.362 0.718)*	*0.8949 (0.798 1.012)*
1989–1992					*0.8987 (0.833 0.948)*	*0.8177 (0.602 0.995)*	*0.8094 (0.664 1.018)*
1993–1997					*0.983 (0.924 1.025)*	*0.6709 (0.465 0.860)*	*0.709 (0.594 0.857)*
1998–2008					*1.035 (0.953 1.102)*	*0.9727 (0.717 1.182)*	*0.626 (0.508 0.781)*
Winship Trites w J/T				*9912 (9260 10553)*			
1984–1988					*0.8748 (0.838 0.906)*	*0.6204 (0.525 0.733)*	*0.9144 (0.814 1.035)*
1989–1992					*0.9098 (0.858 0.952)*	*0.8312 (0.719 0.949)*	*0.8238 (0.694 1.007)*
1993–1997					*0.9908 (0.927 1.044)*	*0.7379 (0.615 0.878)*	*0.7003 (0.589 0.861)*
1998–2008					*1.0448 (0.967 LBR)*	*0.9992 (0.855 1.148)*	*0.6344 (0.532 0.788)*

Adult and juvenile survival scalar, maturity scalar and fertilities derived from a representative selection of Steller sea lion (*Eumetopias jubatus* (Schreber, 1776)) models. Italicized values are parameters that the models estimated. For the % change multipliers under the Reproduction and Survival Senescence Linked (RSSL) models, we used mean fertilities and adult survival over ages and time periods because these were non-linear functions of age and time. 95% confidence limits calculated via the likelihood profile method are in parenthesis where LBR indicates the Limit of Biological Realism where for example, the adult survival scalar could not drop below a particular level that would result in negative adult survival at any age. The non-RSSL models multiplied percentage change scalars directly with the Leslie matrix elements to affect changes over time while the RSSL changed *S_x_* which consequently changed the Leslie matrix elements. HFYS coefficients were taken directly from Holmes et al. (2007). “w J/T” indicates models that included the Juvenile/(Juvenile+Adult) ratio data in fitting the model.

## Discussion

The declines of pregnancy rates with age among harbor seals, northern fur seals and Steller sea lions appear to be closely associated with concomitant declines in survival probabilities. This was most evident for the fur seals (Fig. 4c), and less so for the harbor seals (Fig. 6c) and Steller sea lions (Fig. 5c). However, the fact that we did not have to make any adjustments to fit the derived reproductive rates (i.e., the product of survival and maturity curves) to the sea lion pregnancy rate data suggests there is a close relationship between survival and reproduction for Steller sea lions. The upward adjustment to the fur seal maturity logistic curve was no doubt partially due to using pregnancy rates instead of ovulation rates as our measure of maturity, since pregnancy rates never reach 100% as a true measure of maturity would. Regardless, the small amount of adjustment we applied (Table 3) for all three species supports the hypothesis that reproductive senescence is closely associated with the general physiological decline associated with survival senescence as opposed to reproductive senescence being unlinked to survival senescence as found in human menopause. This makes sense biologically because reproduction would likely be one of the first things an individual forgoes to survive as senescence takes hold at the end of an individual's life (as it is in cases of other physiological stresses such as food limitation or sickness, e.g., [46], [47]).

Data on reproductive senescence is difficult to attain in the field and is generally unavailable for all but the most intensively studied animals. Pinnipeds however tend to be extensively studied because they are either commercially valuable (fur and blubber), compete for commercially valuable fisheries resources, or fall under the “charismatic megafauna” category which often imparts official or implied social importance. Research that has found senescence in pinnipeds has often been part-and-parcel of larger programs to construct life tables to run population dynamics models and address problems related to pinniped commercial value, fisheries or conservation [27], [35], [36].

Including reproductive senescence in models of pinniped population dynamics is likely applicable to many other species, but not all. Of two relatively recent population dynamics models for Steller sea lions, one included reproductive senescence [5], while the other [42] did not. Deciding whether or not to include senescence in a model will depend in part on the question the model is designed to explore and the sensitivity of the output to the added model complexity. Including reproductive senescence does imply increased model complexity. However, incorporating important life history traits should improve model fit and hence the explanatory power and predictive potential of the model.

Our Reproduction and Somatic Survival Linked models showed a tendency for the pregnancy curves (i.e., survival×maturity—Figs. 4–6) of all three of our pinniped species to part ways with the data as they aged. This partly reflects the considerable variability in vital rates that have been estimated from the small number of older animals examined in the field. This was particularly the case for the Steller sea lions where we fit to truncated data (at age 20) and extended the predicted curve to age 30 y. In this particular case, extending our predictive curves in such a manner should not pose a problem when applying the models because only a small percentage of the population remains alive at these older ages, but it may also help to explain why our model did not perform as well as the Holmes et al. (2007) model when fitting to count data and the Juvenile/(Juvenile+Adult) count ratio data.

The best data for demographic studies come from longitudinal mark-recapture studies conducted over generations, but would require considerable investment of time and money. The data we used to parameterize our models came from cross-sectional studies (which are the next best alternative) that occurred during the late 1970's and early 1980's. Survival and fertilities for example were calculated by knowing the numbers of animals shot of different ages and whether or not the animals taken were pregnant. However, the assumptions invoked to derive such vital rates from shot samples [48] (i.e., the population was stable when sampled and all ages had an equal probability of being shot) were likely violated to some extent. Additionally, incorporating the variability of the vital rate curves into the population model (such as in a hierarchical Bayesian setting) would have required proper estimates of variability for the vital rate curves (such as could be obtained for the Weibull survival curve fit to raw data). In addition, it would have also required data to estimate the vital rates of the older age classes which were pooled in some cases (Figs. 5c and 6c).

Of the three pinniped species we examined, we were most confident in the estimates of fur seal survival and fertilities derived from animals shot during the pelagic collection years from 1958–1974 (n = 16,242 females) when numbers of pups produced on St. Paul Island were relatively stable (Fig. 3), as were presumably the number of juveniles and adults. However ages 8 years and under were incompletely represented [49], which meant determining their survival rates using a complex set of assumptions and comparisons with male survival rates as detailed in Lander [31]. Vital rates for the other two pinniped species were obtained by shooting 250 Steller sea lions and 574 harbor seals at rookeries and haulouts in Alaska, but the degree to which the assumptions associated with these cross-sectional collections may have been violated are unknown. Our results must therefore be interpreted with this in mind. Longitudinal mark-recapture studies are underway to estimate life tables for northern fur seals and Steller sea lions (e.g., Hastings et al. [50]) that should ultimately improve the utility of our models.

In general, the fits of the RSSL to the count data of pups and non-pups were excellent and yielded the most parsimonious models. For most populations of pinnipeds, counts of pups and non-pups tend to be the only data available to fit vital rates to. In such cases the RSSL model would be a good choice to investigate changes in vital rates over time when vital rate data indicate a close link to reproductive and survival senescence.

The inability of the RSSL model to fit the count data and ratio of juveniles to total non-pup Steller sea lions could be interpreted to mean there is in fact no link between survival and reproduction. However, the poor fit might also be explained by the life table for Steller sea lions having been calculated from samples taken just prior to a steep population decline in the Gulf of Alaska and Aleutian Islands. Hence, the relationship between survival and reproduction may have differed before, during and after the decline. It is also possible that the relative relationship between survival and fertilities changed or became decoupled during the sea lion population decline (in keeping with the conclusion of Holmes et al. [5] that adult survival increased as fertilities declined). Such changes could occur from density dependence in the vital rates [51] or environmental changes affecting vital rates unequally. Such a decoupling at older ages between fertility and survival would make it difficult for the RSSL model to track data sets with multiple types of observed population metrics. We therefore echo the recommendation and warning of Holmes et al. [5] that more complex demographic data beyond counts of adults and pups can be important in determining vital rate changes that drive population trajectories. Hence, it is possible that static linkages relating vital rates may fail to track changes in population dynamics when population trajectories deviate significantly from the period when data are collected.

A more subtle explanation for the inability of our model to do a better job fitting to the ratio of juveniles to non-pups data may lie with the ratio reflecting a change in behavior rather than a change in numbers. A significant change in the ratio of non-pups to juvenile sea lions might correspond with a change in environmental conditions such as a change in the prey field that could result in adults or juveniles spending more or less time resting on shore between feeding trips. Similarly, an increase in the proportion of juvenile Steller sea lions on shore might reflect an increase in the proportion of juveniles that have not weaned and are staying with their mothers for an extra one or two years. Thus, the juvenile ratio may only be meaningful if it reflects a change in numbers alive rather than a change in behavior.

The ability of our RSSL model to fit the Steller sea lion data better than the Holmes et al. [5] “initially increasing” model is interesting. Trites and Larkin [3] concluded that the populations of Steller sea lions in the early 1970s were not stable but were increasing until the 1980s. Boyd [4] similarly showed an increase in Steller sea lions prior to the decline in the 1980s using a demographic model. The performance of the RSSL model may indicate a real link between adult survival and reproductive ability given that these were the vital rates presumed by the “natural” or unadjusted RSSL model. However, the superior fit of our model rests on fitting 5 initial data points collected prior to 1980 and could be influenced by a paucity of data at the beginning of the population decline.

The fit of our RSSL models, particularly those for the fur seals and harbor seals, may look good even if no linkage exists between birth and survival rates because the number of parameters to the number of observed data points was still relatively high (a caveat that is true for all of our models). In addition, relatively subtle changes in model structure may not make much difference in fit because of the variability of the data and the influence that other unknown factors could have on the year to year variability in numbers. A number of different model structures might therefore be flexible enough to produce similar fits to the data. This might be particularly true for the fur seal data (where the pup counts appear to vary widely around general alternating trends of stability and decline) and in the harbor seal data (where the ratio of estimated parameters to data points was particularly high). In fact, we explored a number of variations on the RSSL theme, estimating one or more of the maturity scalars over time, or by forcing an initial stationary population by changing fertility only, and found very little difference in the results.

The birth and survival rates estimated by the RSSL and literature based models depended upon the structure of the models, while the count data that the models were fit to remained the same (Tables 5–7). For the northern fur seals (Table 5), the RSSL model and the alternative model based on the Lander [31] data gave different parameter values and showed different trends over time in some vital rates, though the fits to the count data were quite similar. The differences indicate that multiple model structures can provide equally compelling fits, if not equally parsimonious fits. Parsimony, however, says nothing about how accurately the models reflect biological events. In fact, the two types of models we compared often indicated opposing biological phenomena. For example the RSSL model predicted a ∼3% decrease in fertilities of northern fur seals between 1975 and 1984, while the Lander model predicted an increase of ∼12% (Table 5).

For the harbor seal (Table 6), fertilities varied considerably between two models, and none of the other models we explored (but have not shown) for this species displayed similar fertility patterns suggesting that the data were not particularly informative. The similarity of the adult survival across models does however suggest that these vital rates may be real for the harbor seals. However, the similar fit of the two models using very different fertilities and juvenile survival rates again implies that model structure was influential. Not having pup or juvenile count data in this case probably confounded the model's ability to differentiate between births and juvenile survival. In addition, both the harbour seal (e.g. 1985–1992 juvenile survival, Table 6) and fur seal (e.g. 1985–1994 adult survival, Table 5) model parameter estimates ran up against the boundaries we imposed for biological realism—and the confidence intervals for the harbour seal juvenile survival adjusters were also relatively large. Our results for northern fur seals and harbor seals show the value of having multiple types of data to create a more dynamic solving surface and definitive minimization, if such modeling methods are to be used to uncover the causes of population fluctuations.

The Steller sea lion model outputs (Table 7) tended to be more consistent over time within model types, and differences between model types were more subtle. The addition of the juvenile to total non-pups ratio changed the juvenile survival and fertilities for the Holmes et al. [5] model, but not the RSSL model. The other previously published models [32], [42] had trends across vital rates that were similar to those of the Holmes et al. [5] model.

Our models incorporated only process error and did not address observation error in the fur seal, harbor seal and sea lion data. State space models have been developed to incorporate observation error and investigating these models is an obvious next step. These models would likely result in wider confidence intervals on the parameters and would better reflect our knowledge of the system. However, such state space models require that the available data be able to inform and differentiate between the two types of error.

With respect to the current thoughts about the causes of the decline of Steller sea lions in the Aleutian Islands and Gulf of Alaska, all of the models associate the initial declines with moderate reduced adult survival and fertilities. However, the juvenile survival scalar varied considerably between all models indicating that juvenile survival was sensitive to model structure and was not well constrained by the data. Thus, apparent changes in juvenile survival rates should be cautiously interpreted. Interspecies comparisons between Steller sea lions, northern fur seals and harbor seals did not indicate any obvious consistent patterns across model types.

### Conclusions

For models that fit count-based data by adjusting age based survival and reproductive rates over time, we found that associating declining rates of survival and reproduction allowed the number of estimated parameters to be reduced while still achieving a good fit to the count based data. Although this link between survival and reproduction means that an RSSL model does not fit data as closely as models that allow total independence between survival and reproductive rates, it can provide a more parsimonious model. The models we considered for the three species of pinnipeds efficiently fit the counts of pups and non-pups, but had difficulty assimilating other metrics of population structure (i.e., juvenile to total non-pup ratios) due possibly to a disassociation of the initial survival and reproduction link, or the confounding influence of behavior on the relative numbers of different age classes resting and counted on shore between feeding trips.

## Supporting Information

Text S1 **R code including data to recreate the analysis and figures for the northern fur seal example.**
(TXT)
